# Development and testing of a European Union-wide farm-level carbon calculator

**DOI:** 10.1002/ieam.1629

**Published:** 2015-05-11

**Authors:** Hanna L Tuomisto, Camillo De Camillis, Adrian Leip, Luigi Nisini, Nathan Pelletier, Palle Haastrup

**Affiliations:** † European Commission, Joint Research Centre (JRC), Institute for Environment and SustainabilityIspra, Italy; ‡ Food and Agriculture Organization of the United Nations, Agriculture and Consumer Protection DepartmentRome, Italy; § Global EcologicVernon, British Columbia, Canada

**Keywords:** Agriculture, C footprint, Farming practices, Greenhouse gas emissions, Life cycle assessment

## Abstract

Direct greenhouse gas (GHG) emissions from agriculture accounted for approximately 10% of total European Union (EU) emissions in 2010. To reduce farming-related GHG emissions, appropriate policy measures and supporting tools for promoting low-C farming practices may be efficacious. This article presents the methodology and testing results of a new EU-wide, farm-level C footprint calculator. The Carbon Calculator quantifies GHG emissions based on international standards and technical specifications on Life Cycle Assessment (LCA) and C footprinting. The tool delivers its results both at the farm level and as allocated to up to 5 main products of the farm. In addition to the quantification of GHG emissions, the calculator proposes mitigation options and sequestration actions that may be suitable for individual farms. The results obtained during a survey made on 54 farms from 8 EU Member States are presented. These farms were selected in view of representing the diversity of farm types across different environmental zones in the EU. The results of the C footprint of products in the data set show wide range of variation between minimum and maximum values. The results of the mitigation actions showed that the tool can help identify practices that can lead to substantial emission reductions. To avoid burden-shifting from climate change to other environmental issues, the future improvements of the tool should include incorporation of other environmental impact categories in place of solely focusing on GHG emissions. *Integr Environ Assess Manag* 2015;11:404–416. © 2015 The Authors. Published by Wiley Periodicals, Inc. on behalf of SETAC.

**Key Points:**

- The methodology and testing results of a new European Union-wide, farm-level carbon calculator are presented.
- The Carbon Calculator reports life cycle assessment-based greenhouse gas emissions at farm and product levels and recommends farm- specific mitigation actions.
- Based on the results obtained from testing the tool in 54 farms in 8 European countries, it was found that the product-level carbon footprint results are comparable with those of other studies focusing on similar products.
- The results of the mitigation actions showed that the tool can help identify practices that can lead to substantial emission reductions.

## INTRODUCTION

Tackling climate change is a key environmental priority on policy makers’ agenda. The current level of atmospheric carbon dioxide has been estimated to exceed the safe boundaries for human well-being, with potentially disastrous consequences for humanity (Rockström et al. 2009). Agriculture accounts for 15% to 25% of global anthropogenic greenhouse gas (GHG) emissions when indirect emissions from direct or indirect land-use change (LUC) are included (Vermeulen et al. 2012). The main sources of GHG emissions from agriculture are nitrous oxide (N_2_O) from soils as a result of nitrogen (N) fertilization, methane (CH_4_) from enteric fermentation of ruminants and manure management, and carbon dioxide (CO_2_) emissions from fossil fuel consumption and degradation of C stocks in soils and vegetation as a result of changes in soil management and land use.

In the European Union (27 Member States; EU-27), direct emissions of GHG from agriculture accounted for approximately 10% of total emissions in 2011 (EEA 2013). Agricultural GHG emissions in EU-27 as reported to UNFCCC have decreased by approximately 20% between 1990 and 2012 mainly because of a decline in livestock numbers and reduced use of fertilizers and changes in manure management (EEA 2013).

To facilitate reductions of farm-level GHG emissions, appropriate and context-specific policy instruments are needed. These measures are to be coupled with supporting tools enabling to inform low-C farming practices. Many farm-level C footprint calculators have been developed to provide support to farmers in identifying the main GHG emission sources and possible reduction strategies (Colomb et al. 2012; Whittaker et al. 2013). Examples include the Cool Farm Tool (Hillier et al. 2011), CLA CALM Calculator (CLA 2014), Farm Carbon Calculator (FCC 2014), and Cplan Carbon Calculator (Cplan 2014). Our aim was to develop a farm-level C calculator that 1) is suitable for the main farming types in the whole EU, 2) presents the C footprint results both at the farm and product scale, and 3) generates farm-specific mitigation action recommendations. A new C calculator was needed, as none of the existing C calculators filled all these functions at the time when the project was started in 2011.

This article describes the key aspects of the C footprinting methodology underpinning the C calculator as well as the key elements of the method to derive the GHG emission mitigation and sequestration options. In addition, this article presents the overall results of the testing phase of the C calculator conducted on 54 European farms.

## MATERIAL AND METHODS

### The Carbon Calculator

**Figure 1:**
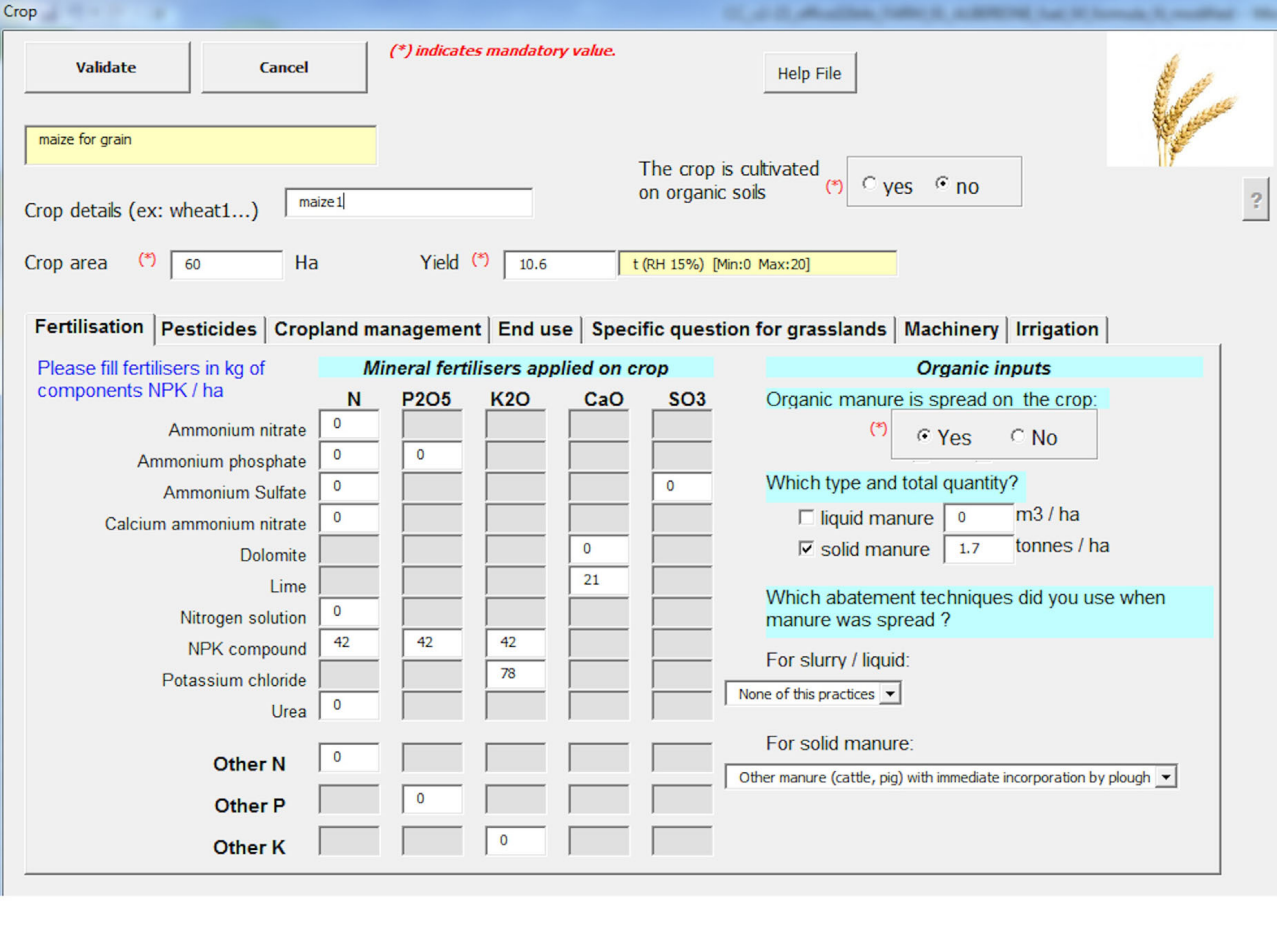
A screen shot of the Carbon Calculator’s user interface for entering data related to crop production.

The Carbon Calculator quantifies life cycle assessment (LCA)-based GHG emissions of a farm and its products. The tool builds on international standards and other technical specifications on life cycle assessment (LCA) and C footprinting (ISO 2006a, 2006b, 2013 GHG Protocol 2011; PAS2050 2011) while striving to be consistent with European Commission’s Environmental Footprinting methods (EC 2013) and the EnviFood Protocol (Food SCP RT 2013). However, the tool does not claim full compliance with any of these technical documents at this stage of development. The tool is designed to be suitable for the most common farm types in the EU-27, and it includes 10 livestock categories (e.g., dairy cattle, beef cattle, meat sheep, pigs, and poultry) and over 150 different crops (including arable, ley, and permanent crops). Rice cultivation, forestry, and fisheries are not included in the tool. The tool is suitable for all farm sizes and farming systems (i.e., organic, conventional, integrated, and conservation farming). The main elements of the Carbon Calculator methodology are described below, whereas a detailed description of the method is in Bochu et al. (2013). The Carbon Calculator has been developed in Microsoft Excel, and Visual Basic for Applications (VBA) is used for creating user forms for data entry (Figure 1). Therefore, specific skills for using Microsoft Excel spreadsheets are not required. The tool has been designed to be used by farmers or farmers’ advisors. The latest version of the Carbon Calculator, the user handbook, methodology guide, and all other project deliverables are available for free download from http://mars.jrc.ec.europa.eu/mars/Projects/LC-Farming. Users are able to modify the formulas and default data when more accurate data is available.

### Carbon footprint assessment

#### System boundaries

The system boundaries for the Carbon Calculator extend from cradle (e.g., nursery and extraction of the raw materials used to manufacture farming equipment and capital goods) to farm gate, including the on- and off-site management of waste produced at farm. Storage and drying are included, but processing of agricultural materials at farm is not included in the system boundaries. The GHG emissions included are CO_2_, CH_4_, N_2_O, and hydrofluorocarbons (HFC). The direct GHG emission sources considered are: CO_2_ emissions from fuel use and burning of crop residues, CH_4_ emissions from ruminant enteric fermentation and manure management, N_2_O emissions from manure and from soils due to use of organic and synthetic N fertilizers, and HFC emissions from leakage of refrigeration gases. In addition, the upstream emissions generated outside of the farm include emissions from the production and transportation of farm inputs, pumping drinking or irrigation water by collective pumping systems, fuel use by contractors for field operations, and N_2_O emissions from NH_3_ volatilization and from N leaching and runoff are incorporated. The user of the Carbon Calculator can choose whether LUC emissions related to purchased feed are included or not. Changes in C stocks in soils (management practices and LUC) and in farmland features (natural infrastructure), as well as GHG emissions avoided through the generation of renewable energy at the farm (whether used on the farm or sold) are quantified but presented separately from other GHG emission results. In addition, the C calculator delivers results in terms of direct primary energy use, water use (irrigation and livestock drinking water), and N balance of the farm.

#### Functional units

The Carbon Calculator quantifies emissions from the whole farm during a year (or production season). Results are presented on the basis of 2 different functional units, i.e., 1) the used agricultural area (UAA) of the farm (tCO_2_-eq/ha UAA), and 2) 1 tonne of each of up to 5 main products as defined by the farmer. UAA includes all cropland, grassland, and permanent crops. Fallow land and conservation areas are included in the UAA. In the case of livestock meat production, the functional unit is 1 tonne of carcass live weight. If the farm produces more than 5 products, the remaining products are allocated to a sixth category named “other products.”

#### Attribution and allocation rules

Allocation between milk and meat is based on protein content, and in the case of eggs and poultry meat allocation is based on energy content. Physical allocation was chosen because it was seen as the first feasible option to implement in the Carbon Calculator when following the allocation hierarchy in ISO 14044 (ISO 2006b) and Environmental Footprint guidelines. Allocation is not done between sheep meat and wool, but all impacts of sheep are allocated to sheep meat. Allocation is not used for crops, but all impacts of crop are allocated to the reported yield output. Meat product output (reported as live carcass weight) is determined based on the weight of the animals sold out during the assessment period. The emissions of the whole cattle during the assessment period are fully allocated to the products (meat, milk, or eggs) delivered by the farm during the assessment period. If the farm does not sell any animals (including young animals) during the assessment period, the C footprint of meat will not be measured by the Carbon Calculator and all emissions will be allocated to the milk sold.

Two options for the end of life management of exported manure are included: manure is spread on another farmland or treated as waste. If manure is spread on another farm, the farm is credited of the avoided emissions from fertilizer production calculated as equal amount of N from mineral fertilizers. The emissions from spreading the manure are allocated to the farm where the manure has been exported. If manure is managed as waste, the emissions from waste management are included in the C footprint of the farm. In both cases, the emissions from transportation of manure are included.

Regarding the use of fuels in farm machinery (excluding machinery use for crop production), electricity, buildings, and other materials used as production inputs (e.g., plastics), the user has to allocate the energy inputs between the products of the farm. Fuel used for field operations is directly attributed to the corresponding crops.

#### Product end-of-life modeling

The emissions from end-of-life management options are modeled only for the major farm-level waste and byproduct streams at the farm level, including manure and plastics. The Carbon Calculator takes into account 3 types of end-of-life treatments of plastics: recycling, reuse (e.g., by returning the plastic containers back to the supplier), and burning.

#### Data sources for emission calculations

The C calculator estimates GHG emission on the basis of a simple activity data–emission factor model. Sources for emission factor data used are presented in Table1.

**Table:** Data sources for emission factors^a^

Emission factor	Data source
CH_4_ emissions	
Enteric fermentation, manure storage (17 different management systems), manure application, manure deposited on pasture land	IPCC 2006; Tier 2
N_2_O emissions	
Manure storage and application N fertilizer use	IPCC 2006; Tier 2
Production and transportation of mineral fertilizers	Weiss and Leip 2012; Wood and Cowie 2004; ADEME 2012; GESTIM 2010; Brentrup and Palliere 2008
HFC emissions	
Refrigerants	ADEME 2012
Inputs	
Purchased feedstuff with LUC emissions	Weiss and Leip 2012
Purchased feedstuff without LUC emissions	ADEME 2012; GESTIM 2010
Pesticides	Green 1987
Seeds, buildings, machinery	ADEME 2012; GESTIM 2010
Plastics	ADEME 2012
Electricity for each country	ELCD 2001
Fuels	Fontelle et al. 2012; ELCD 2001
Collective irrigation	ADEME 2012
Solar energy yields	Nielsen 2011

HFC = hydrofluorocarbons; LUC = land-use change.

a See Bochu et al. (2013) for details.

#### Capital goods and materials

Emissions from construction of buildings and supply of materials are accounted for at the following stages: material production, transportation, construction or implementation, maintenance, and material end-of-life management, including recycling. Emissions from production and assembling of machinery equipment are calculated for each single material component of agricultural equipment. The Carbon Calculator includes a database for emission factors of manufacturing various farm buildings, materials, and machinery. The user indicates the types of materials, buildings, and machinery used at the farm and indicates the percentage of each item used for each product of the farm. In the case of machinery, the tool requires information about the type of the machinery, its age, percentage of the machinery used at the farm, and annual use rate in hours. For buildings, the data required include the type of the building, its age, and surface area. For other materials, the quantities used are reported in mass or volume. Environmental burdens induced by both ordinary and extraordinary maintenance are not taken into account. The yearly emissions of materials, buildings and machinery are calculated by using Equation 1 that is comparable to economic depreciation.

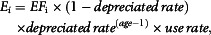
1where *i* = material, building, or machinery; *E*_i_ = emissions from manufacturing of i; *EF*_i_ = emission factor for i; *age* = age (in years) of i; *depreciated rate* = the percentage that the market value of i is assumed to be depreciated during a year; and *use rate* (only for machinery) = overall use of the machinery used at the farm for a specific agricultural products divided by the total use of the machine in terms of its life span.

#### Renewable energy

The following renewable energy sources purchased and used at the farm or generated at the farm (and used at the farm or exported) have been included: firewood, wood chips, solar energy, wind energy, biofuels, electricity from biogas, heat from biogas, and biogas used as gas. Only the GHG emissions avoided by the use of renewable energy in substitution of the use of fossil energy are accounted, whereas the process of production of renewable energy or the manufacturing of the facilities used for generation of renewable energy are not taken into account in the Carbon Calculator. Therefore, the avoided emissions of producing and using renewable energy have to be interpreted with caution. These results are reported separately from other GHG emissions of the farm.

#### Refrigerant emissions

The HFC emissions from refrigeration on a farm are taken into account, including milk tanks in the case of dairy farms, air conditioning in tractors, and air conditioning in offices. The calculation of the HFC emissions from milk tanks is based on reported losses of refrigerant gases or alternatively the losses can be estimated based on Equation 2.

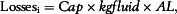
2where i* *= cooling unit (e.g., milk tank, refrigerator, air conditioner); *Losses_i_* = refrigerant losses from the cooling unit (in kg); *Cap* = capacity of the cooling unit (in m^3^); *kgfluid* = 2.1 kg of fluid per m^3^ of storage (Barrualt et al. 2011); and *AL* = percentage of cooling gases lost per year, 15% (Barrualt et al. 2011).

#### Changes in C stocks

The Carbon Calculator reports the changes in C stocks separately from the total results. Changes in soil C stocks are estimated based on IPCC (2006) guidelines (Equation 3).

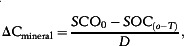
3where Δ*C*_mineral_ = change in C stocks in mineral soils (tonnes C × y^−1^); *SCO*_0_ = soil organic C stock in the previous year of the assessment period (tonnes C); *SOC*_(*o−T*)_ = soil organic C stock at the beginning of the assessment period (tonnes C); *T* = number of years over the assessment period (y) (with *T* = 5 y in the Carbon Calculator); and *D* = time-dependence of stock change factors that is the default period for transition between equilibrium *SOC* values, y (commonly 20 years).

The user can report LUC occurred during the past 20 years at the farm. The following types of land use transformations are considered: forest to cropland, forest to grassland, grassland to cropland, and cropland to grassland. Land conversion from cropland or grassland to forest is not included, as the Carbon Calculator does not take into account forests.

For imported feed, the user can choose whether to include LUC emissions or not by choosing data set with or without the LUC emissions (Table1). The method for calculating the LUC emissions of the imported feed is based on average LUC emission method as explained as Scenario II in Weiss and Leip (2012).

Carbon stocks in perennial crops (e.g., trees, hedgerows, vineyards, or orchards) are taken into account as well as the annual increase of C storage for each category. The following natural infrastructure categories are included: tree natural elements (>5 m high), shrubby natural elements (1–5 m high), and low natural elements (<1 m high). For each category, the C calculator calculates a total C stock, which corresponds to the surface area of each natural vegetation multiplied by the ratio of tonnes of C per ha. In addition to the total C stock, an annual increase of C stock is calculated for each category depending of the quality of the vegetation. The user has the possibility to evaluate the quality in 3 categories: favorable, average, and unfavorable.

The data sources for C stocks in perennial crops are based on French data sources (IFN 2001; INRA 2002) but can be customized if data from other countries are available.

#### Mitigation and sequestration actions

The GHG mitigation and C storage actions as well as the methods for calculating the GHG mitigation potential of those actions are presented in Table2. The selection of mitigation actions included in the tool was based on a review of typical actions recommended in literature and data availability and feasibility to implement in the Carbon Calculator.

**Table 2:** GHG mitigation actions

Action	Target value	Method	Factors impacting GHG mitigation potential after implementing the action
A1: Adjust N fertilizer balance	The farm scale N balance <50 kg N/ha for crops and grassland	The difference between the current N surplus and target value is calculated	Avoided emissions from N fertilizer production Avoided direct and indirect N_2_O emissions due to lower N inputs
A2: Soils covered all the year	100% of cropland covered all the year	Area of bare soil in winter is calculated, and it is assumed that cover crops are planted in this area	Avoided N_2_O emissions due to covering soil in winter compared to bare soil
			Avoided N fertilizer production due to reduced N losses through leaching due to uptake of N by cover crops
			Increased fuel consumption (9 L/ha) due to sowing and harvesting the cover crop
A3: Introduction of legumes in the crop rotation	Proportion of legume crops >20% of cropland (not including grassland)	Assumed that legumes replace some of the land area used for the 3 main crops up to achieving the target value	No need for N fertilizers for legumes
			Reduced N_2_O emissions
			Reduction of N fertilizer use for the following crop by 40 kg/ha
A4: Introduction of legumes in grassland	Proportion of legume crops in grassland >20%	Assumed that the target value is reached without change in quantity of biomass produced	Reduction of N_2_O and CO_2_ emissions due to reduced N fertilization of the grassland (N fertilization is limited to 60 kg/ha)
A5: No-tillage	No-tillage applied to 100% of the cropland and grassland	The area of plowed soil is calculated	Increased C storage in soil
			Increase of soil N-N_2_O emissions by 1 kg/ha
			Reduction of CO_2_ emissions due to reduced fuel consumption (default value of 40 L/ha for no-tillage land used)
A6: Agroforestry in cropland	Area of agroforestry >5% of cropland and temporary grassland	The target value is reached by planting lines of trees on crop- or grassland	C storage increased assuming that C storage in that area is increased by 3 tC ha^−1^ y^−1^
A7: Avoid burning residues	0% of crop residues burned	The current GHG emissions from burning crop residues are quantified	Avoided CO_2_ and CH_4_ emissions from burning crop residues
B1: Reduce methane from enteric fermentation	Digestibility of ruminants’ diet >80% of DE, this can be achieved by changing the feeding of the livestock	The methane emissions from enteric fermentation based on the target value is calculated; the emissions related to higher emissions of the feed production are not taken into account	Reduced methane emissions from enteric fermentation
B2: Change in slurry management system: cover/crust	All liquid slurry storages are covered	The slurry storage without cover is identified and the avoided CH_4_ and N losses due to NH_3_ emissions are quantified	Assumed that 50% of NH_3_ are avoided
			Avoided emissions from N fertilizers produced due to reduced N losses
B3: Biogas production	Manure treated in a biogas reactor	The emissions related to manure management and storage with and without biogas reactor are calculated	Avoided N fertilizer production due to reduced NH_3_ and N_2_ emissions from manure storage
			Reduced N_2_O and CH_4_ emissions from manure storage
C1: Reduction of electricity consumption of the milking systems	10% of electricity for the milking system is saved	It is assumed that 75% of the electricity use of dairy farm is for milking system and it is assumed that this energy use is reduced by 10%	Reduced CO_2_ emissions from electricity production
C2: Reduce engines fuel consumption (test and eco driving)	10% of the fuel for tractors is reduced		Reduced CO_2_ emissions due to reduced use of tractor fuel
C3: Solar panels on suitable buildings	South facing roofs have a solar panel	It is assumed that the south facing roof surface area has a solar panel; the surface is multiplied by yearly irradiation in the country	The avoided emissions of using electricity from grid are calculated
C4: Heat water with solar panels	Hot water used at the farm heated by solar panels	Assumed that water is heated to 65 °C	Calculated the avoided GHG emissions due to use of solar energy instead of fossil energy
C5: Wood boiler	Heating at the farm produced by wood boiler	Assumed that the fossil fuels used for heating are replaced by a wood boiler	CO_2_ emissions avoided from the use of fossil fuels
D1: Implementation of hedges and other landscape elements	>5% of the area of the farm in natural elements	The target is reached by an average quality of C stock in hedges	Increased C storage

GHG = Greenhouse gas, DE = Digestible energy.

To best facilitate decision making of farmers, cost estimations for the following mitigation actions were integrated in the Carbon Calculator: adjusting N fertilizer balance, soils covered year round, reduction of electricity consumption of the milking system, reducing fuel consumption, heating water with a solar panel, and using a wood boiler.

### Farm data

**Figure 2:**
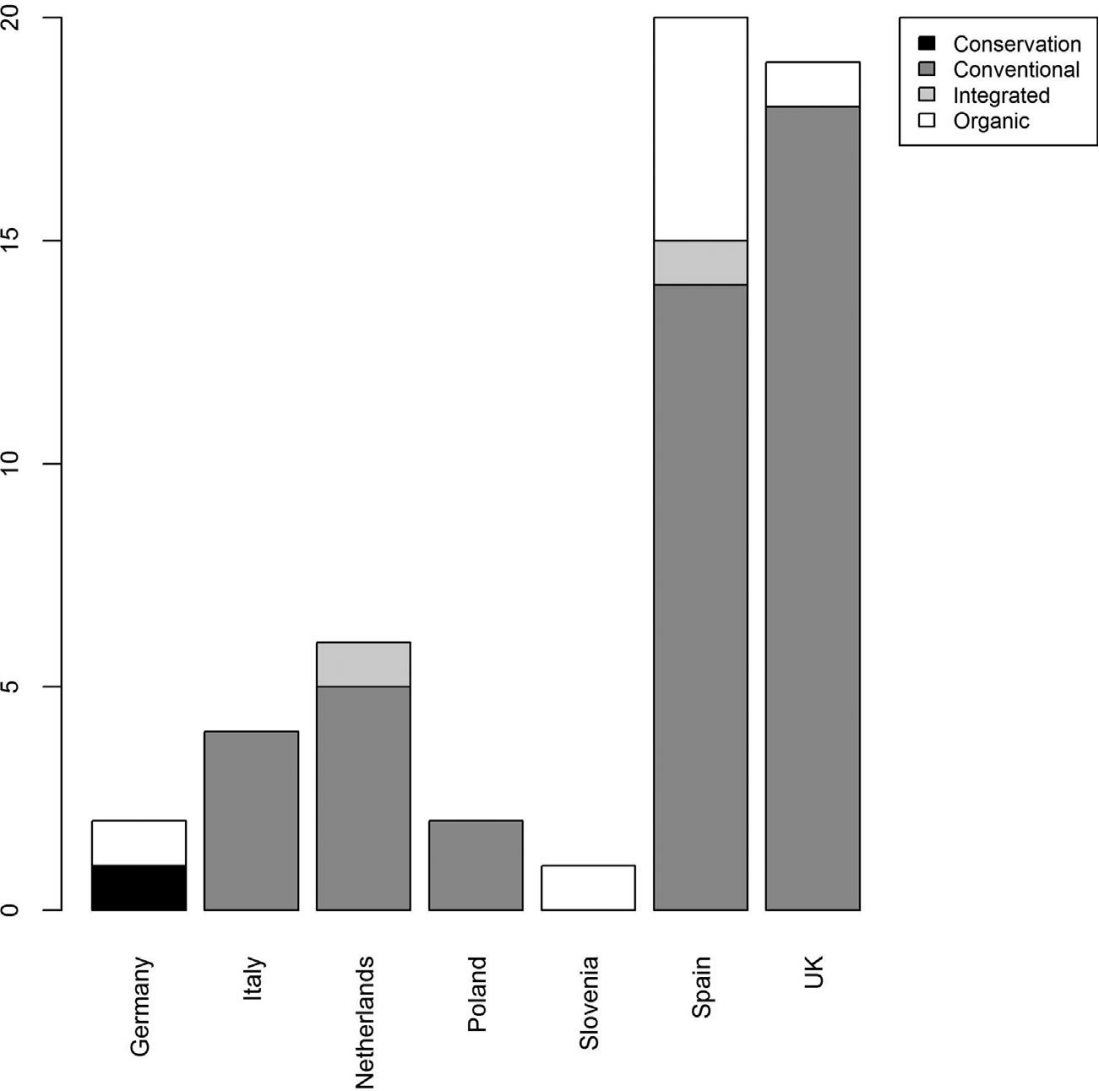
Quantities of farm data sets collected from each country in totals and grouped by farming systems.

Data for the Carbon Calculator were collected from 54 farms in Europe (Figure 2). The study regions were chosen on the basis of their suitability to represent a wide range of environmental zones. The data were collected as a part of a survey that studied the farmers’ willingness to use the C calculator (except the data from Italy) (Elbersen et al. 2013). The Carbon Calculator was demonstrated to farmers before they were requested to complete a survey regarding their willingness to use the tool. The farmers were asked to provide farm data for the Carbon Calculator. At approximately 90% of the farms, the data was collected by a study assistant, and only at approximately 10% of the farms the questionnaire was filled by a farmer without the presence of a member of the research team. The data was collected from farm records when possible. If the data was not available from records, estimates by the farmer or a farmer’s advisor were used. In total, 170 farmers were approached in 8 EU countries (i.e., Denmark, Germany, Spain, Netherlands, Poland, Slovenia, Sweden, and United Kingdom) as a part of the survey. Of these, 50 farmers provided data for the Carbon Calculator. In addition, data from 4 Italian farms were collected in a later stage.

### Statistical analyses

R software was used for the statistical tests. As the data was not normally distributed, the nonparametric Spearman’s rank correlation (Hollander and Wolfe 1973) between the C footprint of the farms and the absolute and relative GHG mitigation potential were assessed.

## RESULTS

### Carbon footprints

The range of the C footprints of the farms in the data set varied from 0.75 tCO_2_-eq/ha up to 63.84 tCO_2_-eq/ha. The C footprint of livestock farms in the data set varied from 1.17 to 63.84 and crop production farms from 0.75 to 6.44.

**Figure 3:**
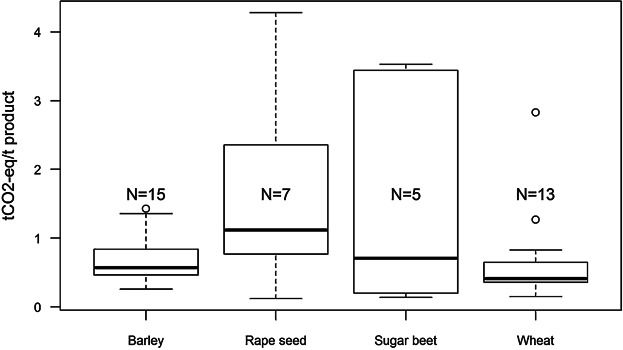
Carbon footprint results of crops (tCO_2_-eq per tonne; N, number of farms that had the crop as 5 main products).

**Figure 4:**
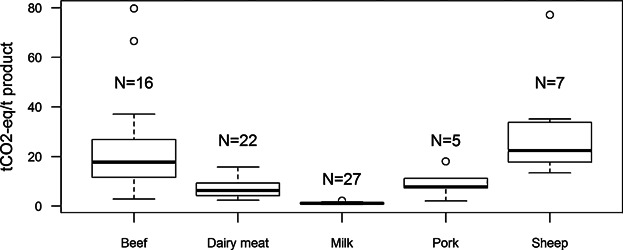
Carbon footprint results of livestock products (tCO_2_-eq per tonne of live weight) when land use change related emissions of purchased feed are not included (N, number of farms that had the livestock type as 5 main products).

The results of the C footprint of products in the data set show wide range of variation between minimum and maximum values (Figures 3 and 4). The wide range of the results especially in livestock sectors is explained by the allocation technique used, which attributes all the emission of the cattle produced during the assessment period to the livestock product output produced during the assessment period. This is discussed further in “*Allocations of emissions*.” The high emissions in some crop farms can be explained by high levels of N fertilizers used at those farms.

### Results of the mitigation actions

**Figure 5:**
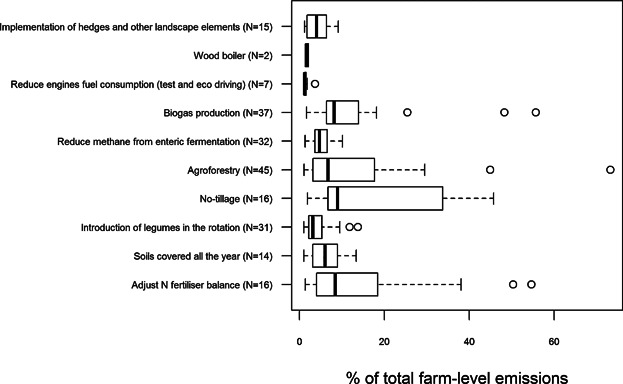
Results of the GHG mitigation potential of the mitigation actions recommended to the farms in the data set (in % of total farm-level emissions reduced; N, number of farms the mitigation action was recommended by the tool and the mitigation potential of the action was >1% of the total emissions of the farm).

The results of the GHG mitigation potential of the mitigation actions are presented in Figure 5. The most commonly recommended mitigation action, “agroforestry,” was recommended to 45 farms in the data set. Other commonly recommended actions were: biogas production, reduce methane from enteric fermentation, and introduction of legumes in the rotation (Figure 5). The mitigation actions with the highest median mitigation rate included no-tillage, biogas production, an adjustment of N balance, and covering soils all the year. The highest maximum mitigation actions in the data set were agroforestry with 73%, biogas production with 56% and adjustment of N fertilizer balance with 55% reduction of the farm’s total emissions. When interpreting the mitigation results presented in Figure 5, it is important to keep in mind that the results are presented at farm level, and therefore, some of the mitigation actions contribute to much higher emissions reduction at product level.

The median cumulative mitigation potential was 28% of the farm emissions, and varied from 1% up to 119%. There was a strong positive correlation (ρ = 0.80 and *p* value < 0.01) between the C footprint of the farms and the cumulative absolute GHG mitigation potential of the farms, whereas a statistically significant negative correlation (ρ = −0.41 and *p* value < 0.01) was found between the C footprint of the farms and the cumulative relative GHG mitigation potential.

## DISCUSSION

### Land use change and soil C stock changes

The Carbon Calculator includes 2 alternative data sets for the emission data of purchased feed, and only one of those data sets includes the LUC emissions. The user has to make the choice between those 2 methods. The choice can have a substantial impact on the C footprint results of farms that use a high quantity of purchased feed. It has been shown that inclusion of LUC emissions can triple or even quadruple the C footprint of livestock products (Weiss and Leip 2012). We found the same effect in our data when we tested the impact of the choice of purchased feed emission data on the results. The effect was strong especially on farms that use soybean feed. It has been shown that emissions of soybeans produced in Amazon region can have even 20 times higher emissions when LUC emissions are included, when compared with situation where LUC emissions are not included (Castanheira and Freire 2013).

In the Carbon Calculator, the emissions related to soil C stock changes were not included in the total C footprint of the farm, but the results were reported separately. This was done because changes in soil C stocks in long term are not possible to estimate unless the multi-annual crop rotations are known (Schrumpf et al. 2011). In the Carbon Calculator, the crop rotation is not known as the production data is provided on annual basis. Therefore, inclusion of the data about whole crop rotations and land management during longer periods in the Carbon Calculator would help to provide more accurate estimates of the long-term changes in soil C stocks and soil N dynamics.

### Allocation of emissions

The wide range of variability of the C footprint results of the livestock products presented in Figure 5 can be explained by the method used in the Carbon Calculator for allocation of emissions to the farm products. The tool allocates all of the emissions related to raising specific livestock species between milk and/or meat sold in that year. For instance, in the case of beef, all emissions related to raising the beef cattle are allocated to the meat sold in that year. Assuming a situation when only a relatively small quantity of animals is slaughtered compared to the total number of animals raised in a certain year, the meat produced receives a relatively high apparent C footprint. Therefore, the allocation of the impacts to the livestock products does not reflect the life cycle impacts, and therefore, caution is needed when the results are compared with other farms or within the same farm between different years. In the development of future versions of the Carbon Calculator, the allocation of emissions to milk and meat needs to be improved. Currently, the C footprint results of livestock products reflect the life cycle-based emissions only in the case where the farm has a balanced distribution of different age groups of animals and sells the same quantity of animals every year.

### Wider impacts

In addition to GHG emissions, the Carbon Calculator reports direct energy and water use, and N balance. To avoid burden shifting from climate change to other environmental impacts, in the future version of the tool the scope of the tool should be enlarged to cover a broader range of impact categories.

It has been shown that C footprint alone is not a sufficient indicator for describing the environmental sustainability of a product (Laurent et al. 2012). Especially, emissions related to toxic substances do not correlate with climate change. However, it has been shown that fossil energy consumption seems to correlate closely with many LCA impact categories in fields of energy production, material production, transport and waste treatment (Huijbregts et al. 2005). Agriculture differs from these fields due to relatively large land use requirements, and therefore, fossil energy input does not necessarily correlate with other environmental impacts, such as eutrophication (Williams et al. 2006).

Furthermore, the Carbon Calculator does not consider issues related to opportunity costs of land use. A farmer may be able to reduce the C footprint of the farm and the products by reducing the farming inputs and production quantities. Consequently, the land use requirement per unit of product may increase. Land is a limited resource, and therefore, optimal land use requires careful planning. It may be justified to use intensive instead of extensive farming practices to save land for provision of other ecosystem services such as biodiversity conservation or wood production (Tuomisto et al. 2012).

### Mitigation actions

Even though the Carbon Calculator does not include all LCA impact categories, the aim was to consider other environmental impact categories and sustainable land use in the choice of mitigation actions included in the tool. For practicality reasons, it was chosen to include only mitigation actions that are relatively easy to implement by farmers. However, the aim was to cover all the most effective mitigation actions, and therefore, a more complex mitigation action “reduce methane from enteric fermentation” was included. The implementation of this action would require help from a farm advisor as it requires making changes in the feeding of livestock.

There is still scope to add more mitigation actions in the future versions of the Carbon Calculator. Because of the high impact of LUC related to purchased soybean feed (Castanheira and Freire 2013), replacing soybeans with local legume crops could be recommended as a mitigation action. In crop production, precision farming technologies and use of controlled release fertilizers and nitrification inhibitors can have substantial impact on reduction of GHG emissions. Precision farming can help to improve nutrient use efficiency and therefore reduce N_2_O emissions and improve yields (de Boer et al. 2011). Nitrification inhibitors have been shown to reduce N_2_O emissions by 40% to 45% from croplands (Pfab et al. 2012), and by 60% to 65% from grazed pastures (Di and Cameron 2012).

In addition to mitigation actions, it is also important to consider actions that maintain or increase C stocks, such as maintenance of permanent grasslands and converting organic soils to permanent grasslands. Restoration of organic soils have been shown to be one of the most effective practices for mitigation of GHG emissions from crop production in area bases, but it also has high mitigation costs (Smith et al. 2008).

The results of the mitigation costs and/or savings of the mitigation actions were not presented in this article because only a few farmers provided the financial data. Based on the data received, many actions provided cost savings because of reduced fertilizer or fuel use. The following mitigation actions are likely result in cost reductions: adjust N fertilizer balance, reduce engines fuel consumption, and reduction of the electricity consumption of the milking system. The Carbon Calculator calculates only the changes in input costs (e.g., fertilizers and energy) but does not take into account investment costs that are required for implementing some of the mitigation actions (e.g., no-till and mitigation actions related to production or utilization of renewable energy) or costs related to changes in the product output quantities (e.g., agroforestry, implementation of hedges, introduction of legumes in the rotation). Other tools for farm management accounting are required for estimating the total costs or savings of those mitigation actions.

### Uncertainties

Currently, the Carbon Calculator does not provide uncertainty ranges for the results. Uncertainties in emission estimates by these types of tools can be high, especially due to wide uncertainties in N_2_O emission estimates. Therefore, implementing uncertainty estimates in the tool would help the interpretation of the reliability of the results. The uncertainties in the C footprint estimates could also be reduced by using process-based modeling of N_2_O emissions (Giltrap et al. 2010; Leip et al. 2011) instead of relying on IPCC emission factors.

The accuracy of the C footprint results produced by the Carbon Calculator depends also on the quality and completeness of data used. Some data (e.g., machinery manufacturing or building data) can be omitted in livestock farms without significant impacts on the results, whereas the accuracy of some other data (e.g., fertilizer and livestock data) is crucial for reliable C footprint estimates.

### Comparison with other C calculators

Colomb et al. (2012) identified 5 C calculators that are suitable to be used worldwide. In addition, their review included over 10 C calculators for both Australia and the United States, 4 tools for the United Kingdom, 3 tools for both Chile and France, 2 for New Zealand, a tool for both Canada and South Africa (wine only), and a tool specific for developing countries. Whittaker et al. (2013) reviewed 11 existing agricultural C calculators suitable for the United Kingdom.

In Table3, we compare the differences of the Carbon Calculator presented in this article with the 5 worldwide tools reviewed by Colomb et al. (2012). The main differences are in the aims of the tools. The EU Carbon Calculator is mainly aimed at being a tool that estimates farm level GHG emissions per unit of land area and allocated to the main products of the farm, and proposes farm-specific mitigation actions. Project evaluation tools (EX-ACT, AFD, and CBP) focus on the change in the emissions as a result of implementing a project instead of aiming at accurate C footprint estimates of the base level. These tools are not only farm level tools but are designed to estimate the effects in a wider region. ALU tool is a reporting tool that aims at estimating the current GHG emissions of agriculture in landscape level.

**Table:** Comparison of carbon calculators suitable for the whole EU^a^

	EU Carbon Calculator	EX-ACT	Cool Farm tool	AFD	CBP	ALU
Formal training required	No	No	No	No	Yes	Yes
Time requirement for an assessment	Medium	Medium	Medium	Low	High	High
Aim of the tool	Reporting (farm calculator)	Project evaluation	Market and product oriented	Project evaluation	Project evaluation	Reporting (landscape calculator)
Field trees, hedges, agroforestry	Yes	No	No	No	Yes	Yes
Forests	No	Yes	Yes	No	Yes	Yes
Capital goods	Yes	Yes	No	Yes	No	No
Fossil fuels and electricity	Yes	Yes	Yes	Yes	No	No
Imported fertilizers and feed	Yes	Fertilizers: yes	Yes	Yes	No	No
		Imported feed: no				
Change in C stocks due to direct LUC	Yes	Yes	Yes	Only deforestation	Yes	Yes
Peat land CH_4_	Yes	Yes	No	No	Yes	Yes
Renewable energy production	Yes	No	Yes	No	No	No
Results types provided	GHG/farm, GHG/ha, GHG/product, GHG reduction by mitigation actions	GHG/ha, GHG/project with comparison with several scenarios	GHG/ha, GHG/product	GHG/farm or territory, GHG/project with comparison with scenarios	GHG/ha, GHG/project with comparison with several scenarios	GHG/farm or territory
Uncertainty accounting	No	Yes	No	No	Yes	No

EU = European Union; GHG = greenhouse gas; LUC = land-use change.

a The information related to the other tools than the EU Carbon Calculator is based on Colomb et al. (2012).

Of the 5 tools evaluated, the Cool Farm tool is the only tool that is focusing on the farm level emissions. The main difference between the Cool Farm tool and the EU Carbon Calculator is Cool Farm tool allows estimating the emissions of 1 product per time only, whereas in the EU Carbon Calculator the emissions of the whole farm and the products of the farm can be assessed simultaneously. In addition, the EU Carbon Calculator proposes farm-specific GHG mitigation actions and shows the mitigation potential of those actions.

## CONCLUSIONS

The Carbon Calculator presented in the article helps identify farm level GHG emission hotspots and mitigation actions that can lead to substantial reductions in C footprints. Therefore, the tool can be used in decision-making processes for designing and implementing climate change mitigation actions for agricultural sector. Carbon calculators are also used by food industry or retailers for accounting and managing emissions along the supply chains.

Many possibilities for improvements of the future versions of the Carbon Calculator were identified during the project. The main shortcoming of the current version of the Carbon Calculator, which could be improved in the future, is related to the attribution of the farm emission to livestock products (as explained earlier in “Allocations of emissions”). Therefore, the C footprint results of livestock products (especially meat) cannot be directly compared between different farms or within the same farm unless the farm has a balanced product output and number of different age groups of animals each year.

The results of crop products can be directly compared between different farms in some extend. Before comparing the results between farms, it is essential to check that each farm has provided all compulsory data requirements and the optional data modules completed in the Carbon Calculator are equal between the farms compared. When the results of farms in different regions are compared, some of the differences in the results may be caused by different soil properties and climatic conditions. Therefore, if the aim is to compare the impact of different farming practices, it is important to compare farms that are located in the same area and have similar soil types. Some error in the results can also be caused by the fact that the current version of the tool cannot address all regional variations in farms (e.g., use of some specific materials or practices).

Further potential improvements include harmonization of the method with Organisational Environmental Footprint guidelines (EC 2013) with the addition of more environmental impact categories. There is also scope with adding more mitigation actions and providing uncertainty assessments. Ideally, if the Carbon Calculator is widely adopted for widespread use in the EU in the future, the possibilities of linking the tool with existing farm-scale databases or other software could reduce the time use for data entry. Furthermore, transferring the tool from Excel to an online application could improve the usability of the tool.
